# Supplementary material for: Identification of photoperiod-induced specific miRNAs in the adrenal glands of Sunite sheep (Ovis aries)
Source: Front Vet Sci. 2022 Jul 22;9:888207. doi: 10.3389/fvets.2022.888207 (PMC9354845; doi:10.3389/fvets.2022.888207)
Supplement: Supplementary file 1 [file Data_Sheet_1.ZIP › Supplementary file/miRDeep2 file.zip!/miRDeep2 file/Novel_Structure/SPLP42A3/Novel_468.pdf]

Provisional ID	:	NC_019479.2_34040
Score total	:	1.5
Score for star read(s)	:	-1.3
Score for read counts	:	0
Score for mfe	:	1.2
Score for randfold	:	1.6
Score for cons. seed	:	
Total read count	:	125
Mature read count	:	125
Loop read count	:	0
Star read count	:	0

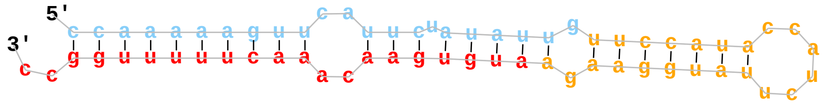

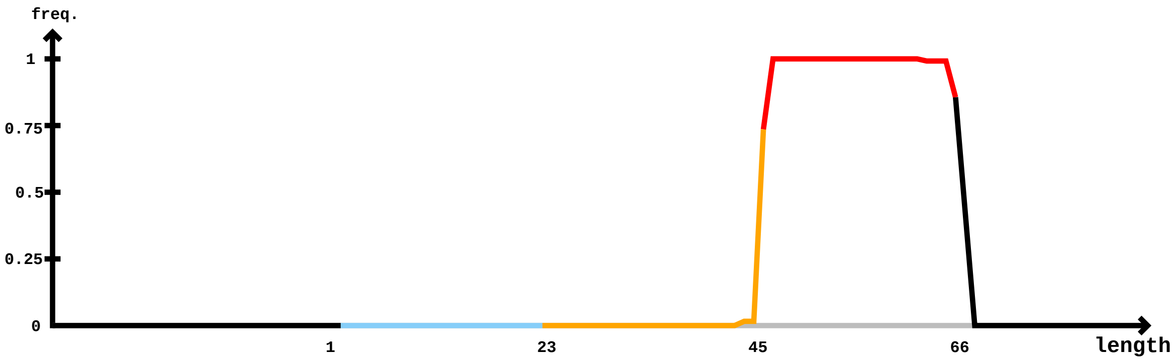

Star Mature

```
5'- aaucaaagacaaaaaaauaucagacuggccaaaaaguucauucuauauuguuccauaccaucuuauggaagaaugugaacaaacuuuuuggccaacccaauauguaauuuaa   -3'    exp
    ................((((..(..(((((((((((((..(((.(((((.(((((((......))))))).))))))))..)))))))))))))..)..)))).........    reads     mm     sample
    ......................................................................Aaaugugaacaaacuuuuu.......................    1         1      seq
    ......................................................................gaaugugaacaaacuuuuuggc....................    1         0      seq
    ........................................................................auCugaacaaacuuuuuggc....................    16        1      seq
    ........................................................................auCugaacaaacuuuuuggcc...................    73        1      seq
    ........................................................................augugaacaaacuuuuuggcU...................    1         1      seq
    .........................................................................uCugaacaaacuuuuuggcc...................    30        1      seq
    .........................................................................uCugaacaaacuuuuuggcca..................    3         1      seq
```
